# Effects of Supervised Cardiac Rehabilitation Programmes on Quality of Life among Myocardial Infarction Patients: A Systematic Review and Meta-Analysis

**DOI:** 10.3390/jcdd8120166

**Published:** 2021-11-27

**Authors:** María Mansilla-Chacón, José L. Gómez-Urquiza, María Begoña Martos-Cabrera, Luis Albendín-García, José L. Romero-Béjar, Guillermo A. Cañadas-De La Fuente, Nora Suleiman-Martos

**Affiliations:** 1 Cenyt Hospital, Avda. Andalucía 2, 29680 Estepona, Spain; mansillachaconmaria@gmail.com; 2 Nursing Department, Faculty of Health Sciences, University of Granada, Avda. de la Ilustración 60, 18016 Granada, Spain; jlgurquiza@ugr.es (J.L.G.-U.); gacf@ugr.es (G.A.C.-D.L.F.); 3 San Cecilio Clinical University Hospital, Andalusian Health Service, Avda. del Conocimiento SN, 18016 Granada, Spain; bego_martos@hotmail.com; 4 Granada-Metropolitano Health District, Andalusian Health Service, C/Joaquina Eguaras 2, 18013 Granada, Spain; lualbgar1979@ugr.es; 5 Statistics and Operational Research Department, University of Granada, Avda. Fuentenueva SN, 18071 Granada, Spain; 6 Nursing Department, Faculty of Health Sciences, Campus Universitario de Ceuta, University of Granada, C/Cortadura del Valle SN, 51001 Ceuta, Spain; norasm@ugr.es

**Keywords:** cardiac rehabilitation, education, quality of life, myocardial infarction, systematic review

## Abstract

Coronary heart disease is the leading cause of death and disability worldwide. Traditionally, cardiac rehabilitation programmes are offered after cardiac events to aid recovery, improve quality of life, and reduce adverse events. The objective of this review was to assess the health-related quality of life, after a supervised cardiac rehabilitation programme, of patients who suffered a myocardial infarction. A systematic review was carried out in the CINAHL, Cochrane, LILACS, Medline, Scopus, and SciELO databases, according to the Preferred Reporting Items for Systematic Reviews and Meta-analysis (PRISMA) guidelines. Randomised controlled trials were selected. Meta-analyses were performed for the Short Form Health Survey SF-36, Myocardial Infarction Dimensional Assessment Scale (MIDAS), MacNew Heart Disease-Health-Related Quality of Life (HRQL) questionnaire, and European Quality of Life-Visual Analogue Scale (EuroQol-VAS) with the software Cochrane RevMan Web. Ten articles were found covering a total of 3577 patients. In the meta-analysis, the effect size of the cardiac rehabilitation programme was statistically significant in the intervention group for physical activity, emotional reaction, and dependency dimensions of the MIDAS questionnaire. For the control group, the score improved for SF-36 physical functioning, and body pain dimensions. The mean difference between the control and intervention group was not significant for the remaining dimensions, and neither for the MacNew Heart Disease-HRQL and EuroQol-VAS questionnaires. Supervised cardiac rehabilitation programmes were effective in improving health-related quality of life, however, there was a potential variability in the interventions; therefore, the results should be interpreted with caution. This study supports the importance of providing care and evaluating interventions via the supervision of trained health professionals, and further randomised clinical trials are needed to analyse the positive changes in mental and physical health outcomes.

## 1. Introduction

Coronary heart disease is the leading cause of death worldwide [1] and its main manifestation is myocardial infarction (MI). This heart disease causes 1.8 million deaths per year, corresponding to 27% of all deaths in Europe [2], and its prevalence is estimated to increase by 18% from 2013 to 2030 [3].

The majority of deaths and hospital admissions are due to MI [4]. In many cases, the symptoms improve with surgical or percutaneous revascularization, reducing mortality [5]. Pharmacological medical treatment also plays an important role in the control of symptoms, especially in nonrevascularizable patients [6].

Despite advances in treatments, after MI with extensive myocardial damage, ventricular dysfunction may appear due to the loss of contractile mass, which is accompanied by the development of heart failure. This fact causes a loss of health-related quality of life due to the inability to perform physical activity when symptoms such as dyspnea, tiredness, and fatigue appear [7]. In addition, up to 25% of patients suffer a deterioration in the quality of life, as well as high levels of anxiety and depression [8]. Therefore, hospital discharge is a critical and challenging time for patients after MI [9]. Coping with a change and readjustment of lifestyle and adherence to new treatments requires support from professionals through continuity of care [10]. These patients are particularly vulnerable to additional cardiac events, and secondary prevention is a priority [7]. This prevention is based on patient education regarding any suspicion of associated symptoms and control of risk factors [7,11].

Among the different intervention strategies, many focus on the control of risk factors [12], and others aim to recover physical activity through cardiac rehabilitation programmes [13,14]. Cardiac rehabilitation is based on measures designed to help patients minimize recovery time after a cardiac event and maximize physical, social, and psychological performance [15]. These interventions aim to promote healthy behaviour in order to alleviate symptoms and reduce limitations [13].

Cardiac rehabilitation programmes appear to be related to the quality of life, being a multifactorial concept that includes the domains of physical, mental, emotional, and social functioning [16]. Patients after MI may have alterations in any of these domains, reducing their well-being in up to 61% of cases [17,18,19].

Some reviews and meta-analyses focused on analysing the effect of unsupervised cardiac rehabilitation programmes by assessing the quality of life in patients with coronary artery disease without counselling and follow-up [20,21,22]. Others analysed programmes that included any core component of cardiac rehabilitation [23], and some programmes even focused only on patients with stable angina [24]. Additional reviews highlighted the improvements in the quality of life in unsupervised home-based cardiac rehabilitation [25], or even analysed parameters such as anxiety and depression [26]. However, few studies analysed the effect of cardiac rehabilitation interventions on health-related quality of life after MI, and no studies focused solely on the analysis of interventions supervised by health professionals. Supervised physical exercise programmes, that include monitoring and counselling by trained health professionals, could positively improve motivation, adherence to healthy habits, and increase exercise tolerance, in order to avoid future cardiac events [7].

Therefore, the objective of this systematic review and meta-analysis was to analyse the effect of supervised cardiac rehabilitation on the improvement of the health-related quality of life in post-MI patients.

## 2. Methods

### 2.1. Design and Search Strategy

A systematic review with meta-analysis was performed following the PRISMA (Preferred Reporting Items for Systematic Reviews and Meta-analyses) statement [27]. The study was registered (ID: 279501) in the PROSPERO database (International Prospective Register of Systematic Reviews). The search was carried out in the CINAHL, Cochrane, LILACS, Medline, Scopus, and SciELO databases. The MeSH terms were used in the following search strategy: “myocardial infarction AND quality of life AND (cardiac rehabilitation OR education) AND randomised controlled trial”. The search was completed in July 2021.

The PICO strategy was used. The population was adults older than 18 years after MI, and the intervention was a supervised cardiac rehabilitation programme (supervised exercise programme, record of level of physical activity, telephone follow-up, or individual counselling). The comparison was addressed to usual care programmes (defined as standard care based on pharmacologic treatment or other non-supervised rehabilitation programmes and may include health education related to diet, education support, or non-structured exercise). The outcome was the measurement of health-related quality of life through validated instruments. Therefore, the research question was: Does a supervised exercise-based cardiac rehabilitation programme influence the health-related quality of life of patients after MI?

### 2.2. Eligibility Criteria and Study Selection

The included studies were: (1) randomised clinical trials, (2) acute myocardial infarction patients, (3) adult samples, (4) hospital or outpatient interventions, (5) health-related quality of life measurements during or after a cardiac rehabilitation programme (baseline data collection before intervention and the follow-up during or after a cardiac rehabilitation programme), (6) rehabilitation programme based on controlled and supervised physical activity, (7) studies published in the last 10 years, (8) not restricted by publication language.

The exclusion criteria were: (1) pilot study or protocols; (2) assessed the health-related quality of life with different interventions, (3) cardiac rehabilitation interventions that did not include physical activity, (4) paediatric patients.

In the selection process, the first two authors independently reviewed the title and abstract of the articles found. Finally, the full text was read. A third author was consulted in case of disagreement.

### 2.3. Data Extraction

The data were recorded by two authors using a data coding manual. A third author verified the data in case of disagreement. The following variables were obtained for each of the articles: (1) author, year and country; (2) design; (3) aims; (4) sample; (5) type of intervention; (6) duration; (7) measuring tool; and (8) main results.

The intraclass correlation coefficient was calculated to assess the reliability of the data coding by the researchers: it was 0.98 (minimum = 0.96; maximum = 1). Cohen’s Kappa coefficient of the categorical variables was 0.97 (minimum = 0.95; maximum = 1).

### 2.4. Quality Assessment and Risk of Bias

The quality and risk of bias of each study were assessed by two authors who collected the data in a table, which were subsequently verified by two other authors. Quality was checked in accordance with the recommendations of the Oxford Center for Evidence-Based Medicine (OCEBM) [28]. The risk of bias of each study was analysed using the Cochrane Collaboration Risk of Bias tool [29].

### 2.5. Data Analysis

Cochrane RevMan Web software was used to carry out the meta-analysis. A total of 18 meta-analyses were carried out, 8 based on the dimensions of the Short Form Health Survey SF-36 (SF-36), 4 based on the dimensions of the MacNew Heart Disease-Health-Related Quality of Life (HRQL) questionnaire, 5 based on the dimensions of the Myocardial Infarction Dimensional Assessment Scale (MIDAS), and 1 based on the European Quality of Life-Visual Analogue Scale (EuroQol-VAS). Heterogeneity was analysed using I^2^ value. Publication bias was assessed with Egger linear regression and sensitivity analysis was performed. Due to the low sample sizes of some of the studies included in the meta-analyses, a random-effects analysis was performed. The questions included in RevMan Web were used for bias analysis. The effect size used was the post-intervention mean and standard deviation provided by the included studies.

## 3. Results

**Figure 1:**
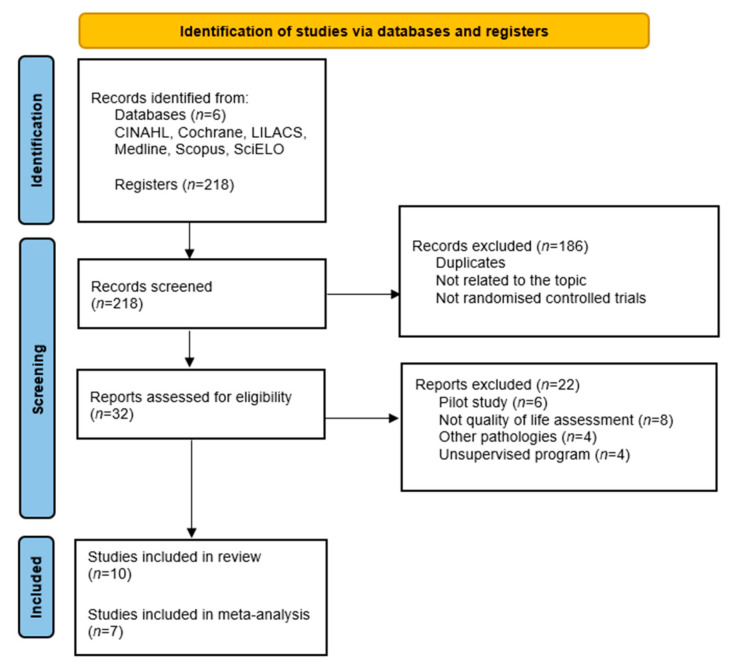
Flow diagram of the publication search process.

After conducting the search 218 articles were found. One-hundred and eighty-six articles were eliminated after reading the title and abstract and removing duplicates. Finally, after reading the full text, the final sample was *n* = 10. The search and selection process is described in Figure 1.

### 3.1. Characteristics of the Studies Included

The total sample size was 3577 patients. All studies were randomised clinical trials conducted in Germany (*n* = 2), and the rest were conducted in Brazil, China, Iran, Italy, Pakistan, Spain, Turkey, and the UK. The main characteristics of all the included studies [30,31,32,33,34,35,36,37,38,39] are listed in Table 1.

The health-related quality of life was measured with the questionnaires SF-36 (*n* = 4), MIDAS (*n* = 2), MacNew Heart Disease-HRQL (*n* = 4), EuroQol-VAS (*n* = 3), European Quality of Life-5 Dimensions (EuroQol-5D) (*n* = 2), and others such as the Self-Rated Health General and Health Questionnaire (*n* = 1). In all studies, the intervention was based on supervised cardiac rehabilitation training, with a duration that ranged from 1 month [30] to 36 months [31]. The exercise included individualised or group programmes, and interventions included cardiorespiratory fitness, such as walking, swimming, balance and strength, and resistance exercises.

### 3.2. Meta-Analysis of the Effect Size of Cardiac Rehabilitation Program on Quality of Life

Studies that provided sufficient statistical information (*n* = 7) were included in the meta-analysis. There were four studies that calculated the effect size in the SF-36 dimensions, and two studies for the MIDAS dimensions, MacNew Heart Disease-HRQL, and EuroQol-VAS.

In the meta-analyses based on the SF-36 questionnaire (*n* = 4), the size of the intervention group was *n* = 1049 patients, while in the control group it was *n* = 1056. Post-intervention means differences were statistically significant for physical functioning and body pain dimensions. In these two cases, the difference in post-intervention means was in favour of the control group. The meta-analysis of the studies using the MIDAS questionnaire had a sample of *n* = 113 in the control group and *n* = 110 in the intervention group. In this questionnaire, the difference was statistically significant for physical activity, emotional reaction, and dependency dimensions in favour of the intervention group. Finally, the differences in means were not significant of the MacNew Heart Disease-HRQL dimensions or to EuroQol-VAS. The effect sizes of each questionnaire are shown in Table 2. Forest plots and the risk of bias are shown in Figure S1–S4 in the Supplementary Materials.

## 4. Discussion

The purpose of this systematic review and meta-analysis was to assess health-related quality of life after a supervised cardiac rehabilitation programme in patients post-MI. The rehabilitation programmes analysed included a supervised exercise programme with a record of the quantity of physical activity, telephone follow-up, or individual counselling. In the intervention group, the results from the MIDAS questionnaire showed an improvement after supervised cardiac rehabilitation in physical activity, emotional reaction, and dependency dimensions, compared to the control group. Other studies found similar results with significant improvements in the intervention group in physical dimension although there was no significant change in mental and emotional dimensions [40]. Normally, patients in the cardiac rehabilitation programmes exercise more frequently and for longer periods and have more information about the benefits of exercise on risk factors, this fact significantly improved health-related quality of life [41,42].

The results from this study indicated improvements in the control group in the dimensions of SF-36 physical functioning and body pain. Other authors found improvements in the control group in all dimensions, except for the role of emotional body pain and vitality [22,43].

In this meta-analysis, we found no improvements after the intervention in health-related quality of life in any other dimension or measurement tool for the intervention group. As corroborated by another meta-analyses, after analysing supervised and non-supervised, exercise-based cardiac rehabilitation, no statistically significant difference, between groups were found for MacNew Heart Disease-HRQL questionnaire [22]. Additionally, other studies corroborated these facts by not finding significant differences between groups [44,45] or by only finding improvements in physical functioning and general health [43,46] or body pain [44] of the SF-36 dimensions.

It seems that the quantity of physical activity performed is closely linked to the health-related quality of life in physical and emotional terms [47]. Therefore, the greater the frequency and duration of the physical activity programmes, the higher the results in the score of each dimension of the SF-36 and MIDAS questionnaires, thus leading to an improvement in health-related quality of life [48,49]. In addition, previous research showed that the early initiation of low-level exercise before discharge from hospital was safe to perform in patients after MI, leading to a significant improvement in exercise tolerance [50,51,52]. Therefore, early exercise led by trained health professionals could positively increase the motivation, which could be translated into increased adherence and tolerance in order to improve health status [51]. Sustained physical activity could also be a key to the quality of life, as well as determining whether the dose and high levels of intensity in the exercise development would be even more beneficial [42,53].

Furthermore, patients who experience MI are more likely to have negative emotional effects that lead to a deterioration of health-related quality of life [54], and thus leaving the treatment and preventing healthy habits. Anxiety and depression are commonly experienced after MI and could persist for months or even years. This fact could also affect access and adherence to rehabilitation programmes; therefore, the early implementation of cardiac rehabilitation programmes could be disrupted [55].

The preventive effects of physical activity, including properly prescribed strength training, are safe and effective in patients with cardiovascular disease [56,57]. Physical training after a cardiac event is essential for improving patient outcomes, as reflected in the recommendation of the American Heart Association [58]. However, this vulnerable population often only receives secondary prevention strategies based on health education, and exercise-based interventions are provided without supervision by health professionals with specific training in this area [59]. Furthermore, few studies include health-related quality of life as an outcome measure when evaluating the effects of cardiac rehabilitation [45,60]. In this study, we found little evidence about the type of intervention, duration of effects over time, or setting (home or centre-based exercise interventions) associated with a true improvement. Providing interventions based on educational support, follow-up and counseling, and supervision by trained health professionals is strongly supported, in order to to improve functional status and health-related quality of life. Developing more randomised clinical trials in different settings, timing, intensity, the type of exercise, and quantity of physical activity could provide evidence for the positive effects on mental and physical health.

### Limitations and Further Research

The present study had several limitations. First, the population included in many of the studies was very small. On the other hand, the interventions were relatively short in time. Furthermore, few studies analysed the adherence to the intervention, hence a compromised control programme could yield different results.

There is also a potential variability due to the types of settings, characteristics of the intervention, follow-up time, and modality (individualised programmes or by groups). Therefore, although the research aim was to analyse the effect of supervised cardiac rehabilitation on the improvement of the health-related quality of life after myocardial infarction, the heterogeneity of approaches adopted may influence the study findings.

Supervised cardiac rehabilitation programmes are effective for improving health-related quality of life. Health policymakers should improve cardiac rehabilitation programmes, promoting supervision by health professionals, with specific training in this area to generate better public health outcomes [61].

Providing more individualized perspectives offers opportunities to measure the health benefits of interventions in terms of survival and quality of life [22]; thus, more clinical trials with larger sample sizes and longer follow-up are needed. In addition, it would be useful to conduct in-depth studies on the adherence to programmes with motivational interventions, such as gamification [62] or coaching interventions [63].

## 5. Conclusions

In the meta-analysis, the effect size of the cardiac rehabilitation programme was statistically significant in the intervention group for physical activity, emotional reaction, and dependency dimensions of the MIDAS questionnaire. For the control group, the score improved in the dimensions for SF-36 physical functioning and body pain. The mean differences between the control and intervention groups were not significant for the remaining dimensions, for MacNew Heart Disease-HRQL, or for EuroQol-VAS questionnaires. Despite finding improvements after cardiac rehabilitation programmes, few studies analyse the effect of a programme supervised by health professionals with the improvement of health-related quality of life as the main objective. More clinical trials with larger sample sizes and longer follow-ups are needed, as well as interventions that support adherence and participation in these programmes.

## Figures and Tables

**Table 1:** Characteristics of the included studies (*n* = 10).

Author, Year, Country	Design/Aims	Sample	Intervention	Duration	Questionnaire	Main Results M (SD)	EL/RG
Campo et al., 2020 [32], Italy	RCT To establish the benefits of an early, tailored and low-cost cardiac exercise intervention	*N* = 235 Mean age 76 years Male 77% *n* GC = 117 *n* IG = 118	CG: usual care IG: supervised sessions (1, 2, 3, 4 months after discharge) + home-based exercise (30–40 min session)	4 months	EuroQol-VAS EuroQol-5D	**Baseline CG/IG** *EuroQol-VAS* 65 (50–80 points)/65 (55–80 points) *EuroQol-5 D* Pain/Discomfort: Extreme-Moderate 15.5%/14.5% Anxiety/Depression: Extremely-Moderate 21%/23% **1-year follow-up CG/IG** *EuroQol-VAS* 65 (50–80 points)/75 (70–87) *EuroQol-5 D* Pain/Discomfort: Extreme-Moderate 17%/11% Anxiety/Depression: Extremely- Moderate 24%/13%	1b/A
Ebrahimi et al., 2021, [33], Iran	RCT To assess the effect of peer education on quality of life and self-care behaviour	*N* = 70 Mean age 55.66 yearsMale 65.71% *n* CG = 35 *n* IG = 35	CG: usual care IG: two one-hour training sessions	4 weeks	MacNew Heart Disease-HRQL	After intervention, the score improved in all quality-of-life dimensions (emotional functioning, physical functioning, and social functioning) (*p* < 0.05)	1b/A
Jaureguizar et al., 2016, [34], Spain	RCT To determine the impact of the type of exercise on quality of life	*N* = 72 Mean age 58 years Male 85% *n* CG = 36 *n* IG = 36	CG: usual careIG: high intensity interval training (40 min per session, 3 days per week). Total of 24 sessions	8 weeks	SF-36 MacNew Heart Disease-HRQL	**Baseline CG/IG** *SF-36* Physical functioning 73 (24)/78 (15) Role physical 51 (43)/49 (42) Body pain 67 (30)/72 (23) General health 58 (19)/58 (18) Vitality 62 (18)/57 (19) Social functioning 83 (22)/82 (19) Role emotional 73 (38)/48 (44) Mental health 70 (20)/64 (17) Self-reported health status 3 (1)/3 (1) Physical health index 43 (11)/47 (8) Mental health index 48 (12)/41.0 (12.4) *MacNew Heart Disease-HRQL* Emotional domain 5.5 (1.1)/5.3 (0.9) Physical domain 5.6 (0.9)/5.5 (1.0) Social domain 5.7 (0.9)/5.6 (0.9) Global domain 5.5 (0.9)/5.3 (0.9) **8-week follow-up CG/IG** *SF-36* Physical functioning 77 (23)/83 (16) Role physical 59 (44)/54 (45) Body pain 73 (25)/74 (25) General health 62 (22)/63 (19) Vitality 67 (18)/63 (23) Social functioning 83 (22)/89 (17) Role emotional 75 (40)/73 (36) Mental health 73 (22)/73 (18) Self-reported health status 2 (1)/2 (1) Physical health index 46(12)/47 (7) Mental health index 50 (14)/49 (11) *MacNew Heart Disease-HRQL* Emotional domain 5.7 (1.1)/5.8 (0.9) Physical domain 5.9 (0.9)/5.9 (1.0) Social domain 6.0 (0.8)/6.0 (0.9) Global domain 5.8 (0.9)/5.8 (0.9)	1b/A
Mayer-Berger et al., 2014, [31], Germany	RCT To evaluate the efficacy of a long-term secondary prevention programme following inpatient cardiovascular rehabilitation	*N* = 600 Mean age 49.2 years Male 89.15% *n* CG = 329 *n* IG = 271	CG: usual care IG: inpatient cardiac rehabilitation, one rehabilitation session, and regular telephone reminder (3 units of exercise per day, 30–60 min per unit)	36 months	EuroQol-VAS EuroQol-5D HADS	**Baseline CG/IG** *EuroQol-VAS* CG: 61 (18.5)IG: 61.1 (18.5) *EuroQol-5D* CG: 75.9 (17.3) IG: 76 (16.8)*HADS anxiety* CG: 7.7 (4.2) IG: 7.5 (4.1) *HADS depression* CG: 6.0 (4) IG: 5.6 (3.8) **36-month follow-up CG/IG** *EuroQol-VAS* CG: 64.5 (18.9) IG: 72.2 (18.4) *EuroQol-5D* CG: 75.5 (18.7) IG: 78.6 (16.9) *HADS anxiety* CG: 7.4 (4.8) IG: 6.6 (4.1) *HADS depression* CG: 5.7 (4.3) IG: 4.6 (4.1)	1b/A
Peixoto et al., 2015, [30], Brazil	RCT To evaluate the influence of an early cardiac rehabilitation programme on HRQL and functional capacity	*N* = 88 Mean age 56 years Male 70% *n* CG = 43 *n* IG = 45	CG: usual care IG: early intensive cardiac rehabilitation programme (4 times per week)	1 month	MacNew Heart Disease-HRQL	**1-month follow-up CG/IG** *MacNew Heart Disease-HRQL* Social domain 5.2 (1.2)/5.7 (1.0) Physical domain 4.9 (0.9)/6.1 (0.7) Emotional domain 4.9 (1.2)/6.0 (0.7) Global domain 5.2 (1.0)/6.1 (0.6)	1b/A
Ul-Haq et al., 2019, [35], Pakistan	RCT To find out the effectiveness of cardiac rehabilitation in patients with MI	*N* = 195 Mean age 53 years Male 76.92% *n* CG = 96 *n* IG = 99	CG: usual care IG: cardiac rehabilitation programme (counselling and health education, medicine prescription, and follow-up advice)	8 weeks	Self-Rated Health GeneralHealth Questionnaire MacNew Heart Disease-HRQL	**Baseline CG/IG** Self-Rated Health 3.9 (0.07)/3.97 (0.9) General Health Questionnaire 18.71 (4.3)/21.2 (5.5) MacNew Heart Disease-HRQL 3.9 (0.5)/3.6 (1.07) **8-week follow-up CG/IG** Self-Rated Health 4.06 (0.06)/2.3 (0.8) General Health Questionnaire 20.9 (5.2)/7.4 (4.2) MacNew Heart Disease-HRQL 3.8 (0.5)/5.6 (0.5)	1b/A
Uysal and Özcan, 2012, [36], Turkey	RCT To identify the effect of individual training and counselling programme for patients having experienced MI	*N* = 90 Age > 55 45.6% Male 77.8% *n* CG = 45 *n* IG = 45	CG: usual care IG: training and counselling programme (60 min session)	3 months	MIDAS SF-36	**Baseline CG/IG** *MIDAS* Physical activity 14.8 (3.3)/14.3 (4.0) Insecurity 4.1 (3.4)/4.2 (2.8) Emotional reaction 9.2 (3.2)/7.9 (3.7) Social activity 6.5 (2.5)/6.0 (2.4) Dependency 6.7 (2.8)/6.4 (2.5) Concern over medication 1.5 (1.7)/1.0 (1.2) *SF-36* Physical functioning 58.6 (27.9)/57.2 (24.8) Role physical 146.6 (50.4)/145.5 (49.5) Body pain 35.6 (12.3)/37.5 (12.7) General health 47.6 (14.3)/45.0 (14.6) Vitality 37.1 (10.0)/38.5 (10.8) Social functioning 45.5 (16.4)/47.2 (13.8) Role emotional 146.6 (50.4)/137.7 (47.9) Mental health 45.3 (10.4)/45.5 (11.4) **3-month follow-up CG/IG** *MIDAS* Physical activity 3.8 (2.8)/1.9 (2.3) Insecurity 2.5 (2.3)/1.0 (0.8) Emotional reaction 5.1 (2.5)/1.2 (2.4) Social activity 5.1 (2.0)/2.4 (1.3) Dependency 6.7 (2.8)/1.9 (1.5) Concern over medication 1.5 (1.7)/0.2 (0.4) *SF-36* Physical functioning 77.1 (14.1)/87.5 (10.9) Role physical 146.6 (50.4)/177.7 (42.0) Body pain 76.2 (12.4)/83.1 (11.6) General health 52.3 (12.8)/51.2 (12.7) Vitality 51.1 (14.3)/73.5 (14.5) Social functioning 63.6 (10.9)/90.2 (18.6) Role emotional 145.1 (49.8)/197.7 (14.9) Mental health 53.0 (10.6)/77.8 (15.5)	1b/A
Wang et al., 2012, [37], China	RCT To evaluate the effects of a home-based rehabilitation programme in patients with MI in terms of health-related quality of life and psychological status	*N* = 133 Mean age 57.8 years Male 83.4% *n* CG = 65 *n* IG = 68	CG: usual care IG: home-based rehabilitation care (fitness plan, including a home exercise, relaxation plan, and telephone counselling)	6 months	MIDAS SF-36	**Baseline CG/IG** *MIDAS* Physical activity 51.3 (16.5)/55.1 (14.5) Insecurity 37.0 (16.6)/41.1 (16.7) Emotional reaction 38.5 (19.0)/41.7 (21.2) Dependency 39.3 (18.9)/43.4 (22.6) Concerns over medications 40.8 (21.7)/48.1 (23.1) *SF-36* Physical functioning 55.0 (20.0)/50.0 (24.9) Role physical 33.8 (46.2)/31.2 (42.1) Body pain 35.1 (20.2)/30.0 (16.6) General health 43.6 (18.2)/39.2 (20.6) Vitality 46.9 (26.5)/47.1 (23.0) Social functioning 54.8 (20.7)/50.1 (24.8) Role emotional 54.8 (46.9)/46.6 (46.8) Mental health 59.1 (23.1)/57.2 (22.9) **6-month follow-up CG/IGMIDAS** Physical activity 42.6 (12.3)/37.7 (11.2) Insecurity 33.4 (13.8)/28.7 (9.7) Emotional reaction 34.8 (14.4)/30.4 (12.8) Dependency 31.8 (16.6)/27.6 (9.4) Concerns over medications 37.7 (18.0)/29.4 (12.6) **SF-36** Physical functioning 73.2 (13.0)/80.8 (13.7) Role physical 56.2 (46.8)/68.2 (17.3) Body pain 63.5 (14.6)/68.2 (17.3) General health 49.0 (16.2)/57.4 (20.3) Vitality 56.4 (21.7)/66.3 (17.3) Social functioning 65.8 (18.0)/71.3 (21.4) Role emotional 75.9 (39.7)/80.8 (37.9) Mental health 65.4 (20.7)/73.5 (17.1)	1b/A
West et al., 2012, [38], UK	RCT To determine the effect of cardiac rehabilitation, on health-related quality of life in patients following MI	*N* = 1813 Mean age 64 years Male 73% *n* CG = 910 *n* IG = 903	CG: usual care IG: exercise training, health education, and counselling (total 20 h)	8 weeks	SF-36 PGWB	**Baseline CG/IG** *SF-36* Physical functioning 48 (24)/48 (23) Role physical 22 (27)/20 (26) Body pain 73 (27)/74 (26) General health 65 (24)/65 (23) Vitality 45 (24)/45 (24) Social functioning 63 (31)/61 (32) Role emotional 67 (41)/64 (43) Mental health 73 (21)/72 (21) *PGWB domain* Anxiety 19.0 (4.9)/19.2 (4.7) Depression 12.6 (2.8)/12.6 (2.6) Positive well-being 11.3 (3.9)/11.3 (4.0) **12-month follow-up CG/IG** *SF-36* Physical functioning 64 (30)/65 (29) Role physical 67 (33)/69 (31) Body pain 68 (29)/69 (28) General health 57 (25)/58 (25) Vitality 65 (24)/65 (24) Social functioning 79 (29)/81 (28) Role emotional 67 (41)/64 (43) Mental health 76 (13)/76 (13) *PGWB domain* Anxiety 19.8 (4.7)/19.8 (4.4) Depression 12.3 (3.8)/12.3 (3.9) Positive well-being 12.9 (2.7)/13.0 (2.6)	1b/A
Wienbergen et al., 2019, [39], Germany	RCT To compare an intensive prevention programme with usual care after MI	*N* = 281 Mean age 56.5 years Male 81.5% *n* CG = 143 *n* IG = 138	CG: usual care IG: intensive programme (education sessions, telephone visits, and telemetric risk factor control)	12-month	EuroQol-VAS PHQ-9	**Baseline CG/IG** EuroQol-VAS 77.6 (13)/76.4 (15) PHQ-9 3.9 (3.5)/4.4 (3.5) **12-month follow-up** EuroQol-VAS 77.1(14)/78.2 (15) PHQ-9 4.3 (4.2)/3.6 (3.5)	

Note: CG = Control Group; EL = Evidence level; EuroQol-5D = European Quality of Life-5 Dimensions; EuroQol-VAS = European Quality of Life -Visual Analogue Scale; HADS = Hospital Anxiety and Depression Scale; HRQL = Health-Related Quality of Life; IG = Intervention Group; MI = Myocardial Infarction; MIDAS = Myocardial Infarction Dimensional Assessment Scale; PHQ-9 = Patient Health Questionnaire-9; PGWB = Psychological General Well-Being; RCT = Randomised controlled trial; RG = Recommendation grade; SF-36 = Short Form Health Survey SF-36.

**Table 2:** Effect size (post-intervention mean difference).

Tool and Domain	Effect Size (95% CI)	*p*-Value Z Test
Physical functioning (SF-36)	5.88 (0.93, 10.83)	0.02
Physical role (SF-36)	8.97 (−2.92, 20.86)	0.14
Mental health (SF-36)	8.30 (−4.29, 20.88)	0.20
Body pain (SF-36)	3.33 (0.11, 6.56)	0.04
General health (SF-36)	1.96 (−1.59, 5.51)	0.28
Vitality (SF-36)	7.22 (−4.37, 18.81)	0.22
Social functioning (SF-36)	9.98 (−1.61, 21.58)	0.09
Role emotional (SF-36)	11.18 (−13.78, 36.13)	0.38
Physical activity (MIDAS)	−2.75 (−5.41, −0.10)	0.04
Insecurity (MIDAS)	−2.45 (−5.31, 0.42)	0.09
Emotional reaction (MIDAS)	−2.75 (−3.55, −1.95)	<0.01
Dependency (MIDAS)	−4.78 (−5.69, −3.87)	<0.01
Concern over medication (MIDAS)	−4.28 (−11.06, 2.50)	0.22
Emotional domain (MacNew-HRQL)	0.61 (−0.37, 1.59)	0.23
Physical domain (MacNew-HRQL)	0.61 (−0.57, 1.78)	0.31
Social domain (MacNew-HRQL)	0.23 (−0.25, 0.72)	0.35
Global domain (MacNew-HRQL)	0.46 (−0.42, 1.34)	0.31
EuroQol-VAS	4.45 (−2.02, 10.92)	0.18

Note: EuroQol-VAS = European Quality of Life-Visual Analogue Scale; MacNew-HRQL = MacNew Heart Disease-Health-Related Quality of Life; MIDAS = Myocardial Infarction Dimensional Assessment Scale; SF-36 = Short Form Health Survey SF-36.

## Data Availability

Data are available upon request to the corresponding author.

## Supplementary Materials

The following are available online at https://www.mdpi.com/article/10.3390/jcdd8120166/s1, Figure S1: Effect size forest plot using SF-36 questionnaire, Figure S2: Effect size forest plot using MIDAS questionnaire, Figure S3: Effect size forest plot using MacNew Heart Disease-HRQL, Figure S4: Effect size forest plot using EuroQol-VAS.

## References

[1] World Health Organization Cardiovascular Diseases (CVDs). https://www.who.int/en/news-room/fact-sheets/detail/cardiovascular-diseases-(cvds).

[2] Timmis A., Townsend N., Gale C.P., Torbica A., Lettino M., Petersen S.E., Mossialos E.A., Maggioni A.P., Kazakiewicz D., May H.T. (2020). European Society of Cardiology: Cardiovascular Disease Statistics 2019. Eur. Heart J..

[3] Mozaffarian D., Benjamin E.J., Go A.S., Arnett D.K., Blaha M.J., Cushman M., de Ferranti S., Després J.P., Fullerton H.J., Howard V.J. (2015). Heart disease and stroke statistics-2015 update: A report from the American Heart Association. Circulation.

[4] Reed G.W., Rossi J.E., Cannon C.P. (2017). Acute myocardial infarction. Lancet.

[5] Bajaj A., Sethi A., Rathor P., Suppogu N., Sethi A. (2015). Acute complications of myocardial infarction in the current era: Diagnosis and management. J. Investig. Med..

[6] Ali M.A., Yasir J., Sherwani R.N., Fareed M., Arshad F., Abid F., Arshad R., Ismail S., Khan S.A., Siddiqui U.J. (2017). Frequency and predictors of non-adherence to lifestyle modifications and medications after coronary artery bypass grafting: A cross-sectional study. Indian Heart J..

[7] Valaker I., Norekvål T.M., Råholm M.B., Nordrehaug J.E., Rotevatn S., Fridlund B. (2017). Continuity of care after percutaneous coronary intervention: The patient’s perspective across secondary and primary care settings. Eur. J. Cardiovasc. Nurs..

[8] Tully P.J. (2012). Psychological depression and cardiac surgery: A comprehensive review. J. Extra. Corpor. Technol..

[9] Fålun N., Fridlund B., Schaufel M.A., Schei E., Norekvål T.M. (2016). Patients’ goals, resources, and barriers to future change: A qualitative study of patient reflections at hospital discharge after myocardial infarction. Eur. J. Cardiovasc. Nurs..

[10] Stoicea N., You T., Eiterman A., Hartwell C., Davila V., Marjoribanks S., Florescu C., Bergese S.D., Rogers B. (2017). Perspectives of post-acute transition of care for cardiac surgery patients. Front. Cardiovasc. Med..

[11] Stevens S. (2015). Preventing 30-day Readmissions. Nurs. Clin. N. Am..

[12] Weber B., Bersch-Ferreira Â.C., Torreglosa C.R., Marcadenti A., Lara E.S., Da Silva J.T., Costa R.P., Santos R.H., Berwanger O., Bosquetti R. (2019). Implementation of a Brazilian Cardioprotective Nutritional (BALANCE) Program for improvement on quality of diet and secondary prevention of cardiovascular events: A randomized, multicenter trial. Am. Heart J..

[13] Long L., Mordi I.R., Bridges C., Sagar V.A., Davies E.J., Coats A.J.S., Dalal H., Rees K., Singh S.J., Taylor R.S. (2019). Exercise-based cardiac rehabilitation for adults with heart failure. Cochrane Database Syst. Rev..

[14] Grässler B., Thielmann B., Böckelmann I., Hökelmann A. (2021). Effects of different exercise interventions on cardiac autonomic control and secondary health factors in middle-aged adults: A Systematic Review. J. Cardiovasc. Dev. Dis..

[15] Buckley J.P., Furze G., Doherty P., Speck L., Connolly S., Hinton S., Jones J.L. (2013). BACPR scientific statement: British standards and core components for cardiovascular disease prevention and rehabilitation. Heart.

[16] Jolliffe J.A., Rees K., Taylor R.S., Thompson D., Oldridge N., Ebrahim S. (2000). Exercise-based rehabilitation for coronary heart disease. Cochrane Database Syst. Rev..

[17] Kurfirst V., Mokráček A., Krupauerová M., Čanádyová J., Bulava A., Pešl L., Adámková V. (2014). Health-related quality of life after cardiac surgery—The effects of age, preoperative conditions and postoperative complications. J. Cardiothorac. Surg..

[18] Correa-Rodríguez M., Abu Ejheisheh M., Suleiman-Martos N., Membrive-Jiménez M.J., Velando-Soriano A., Schmidt-RioValle J., Gómez-Urquiza J.L. (2020). Prevalence of depression in coronary artery bypass surgery: A systematic review and meta-analysis. J. Clin. Med..

[19] Ejheisheh M.A., Correa-Rodríguez M., Fernández-Aparicio Á., Batran A., Suleiman-Martos N., Schmidt-RioValle J. (2020). Prior percutaneous coronary intervention is associated with low health-related quality of life after coronary artery bypass graft. Nurs. Health Sci..

[20] McGregor G., Powell R., Kimani P., Underwood M. (2020). Does contemporary exercise-based cardiac rehabilitation improve quality of life for people with coronary artery disease? A systematic review and meta-analysis. BMJ Open.

[21] Oldridge N., Pakosh M., Grace S.L. (2019). A systematic review of recent cardiac rehabilitation meta-analyses in patients with coronary heart disease or heart failure. Future Cardiol..

[22] Candelaria D., Randall S., Ladak L., Gallagher R. (2020). Health-related quality of life and exercise-based cardiac rehabilitation in contemporary acute coronary syndrome patients: A systematic review and meta-analysis. Qual. Life Res..

[23] Francis T., Kabboul N., Rac V., Mitsakakis N., Pechlivanoglou P., Bielecki J., Alter D., Krahn M. (2019). The Effect of Cardiac Rehabilitation on Health-Related Quality of Life in Patients with Coronary Artery Disease: A Meta-analysis. Can. J. Cardiol..

[24] Long L., Anderson L., He J., Gandhi M., Dewhirst A., Bridges C., Taylor R. (2019). Exercise-based cardiac rehabilitation for stable angina: Systematic review and meta-analysis. Open Hear..

[25] Shepherd C., While A. (2012). Cardiac rehabilitation and quality of life: A systematic review. Int. J. Nurs. Stud..

[26] Zheng X., Zheng Y., Ma J., Zhang M., Zhang Y., Liu X., Chen L., Yang Q., Sun Y., Wu J. (2019). Effect of exercise-based cardiac rehabilitation on anxiety and depression in patients with myocardial infarction: A systematic review and meta-analysis. Hear. Lung.

[27] Page M.J., McKenzie J.E., Bossuyt P.M., Boutron I., Hoffmann T.C., Mulrow C.D., Shamseer L., Tetzlaff J.M., Akl E.A., Brennan S.E. (2021). The PRISMA 2020 statement: An updated guideline for reporting systematic reviews. BMJ.

[28] Howick J., Chalmers I., Glasziou P., Greenhalg T., Heneghan C., Liberati A., Moschetti I., Phillips B., Thornton H. The Oxford 2011 Levels of Evidence. https://www.cebm.net/2016/05/ocebm-levels-of-evidence.

[29] Higgins J.P.T., Green S. Cochrane Handbook for Systematic Reviews of Interventions. Version 5.1.0. The Cochrane Collaboration. www.cochrane-handbook.org.

[30] Peixoto T.C.A., Begot I., Bolzan D.W., Machado L., Reis M.S., Papa V., Carvalho A.C.C., Arena R., Gomes W.J., Guizilini S. (2015). Early exercise-based rehabilitation improves health-related quality of life and functional capacity after acute myocardial infarction: A randomized controlled trial. Can. J. Cardiol..

[31] Mayer-Berger W., Simic D., Mahmoodzad J., Burtscher R., Kohlmeyer M., Schwitalla B., Redaèlli M. (2014). Efficacy of a long-term secondary prevention programme following inpatient cardiovascular rehabilitation on risk and health-related quality of life in a low-education cohort: A randomized controlled study. Eur. J. Prev. Cardiol..

[32] Campo G., Tonet E., Chiaranda G., Sella G., Maietti E., Bugani G., Vitali F., Serenelli M., Mazzoni G., Ruggiero R. (2020). Exercise intervention improves quality of life in older adults after myocardial infarction: Randomised clinical trial. Heart.

[33] Ebrahimi H., Abbasi A., Bagheri H., Basirinezhad M.H., Shakeri S., Mohammadpourhodki R. (2021). The role of peer support education model on the quality of life and self-care behaviors of patients with myocardial infarction. Patient Educ. Couns..

[34] Jaureguizar K., Vicente-Campos D., Ruiz Bautista L., de La Peña C.H., Arriaza Gómez M.J., Calero Rueda M.J., Fernández Mahillo I. (2016). Effect of high-intensity interval versus continuous exercise training on functional capacity and quality of life in patients with coronary artery disease: A randomized clinical trial. J. Cardiopulm. Rehabil. Prev..

[35] Ul-Haq Z., Khan D., Hisam A., Yousafzai Y.M., Hafeez S., Zulfiqar F., Gul A.M., Hafizullah M., Pell J. (2019). Effectiveness of cardiac rehabilitation on health-related quality of life in patients with myocardial infarction in Pakistan. J. Coll. Physicians Surg. Pakistan.

[36] Uysal H., Özcan Ş. (2012). The effect of individual training and counselling programme for patients with myocardial infarction over patients’ quality of life. Int. J. Nurs. Pract..

[37] Wang W., Chair S.Y., Thompson D.R., Twinn S.F. (2012). Effects of home-based rehabilitation on health-related quality of life and psychological status in Chinese patients recovering from acute myocardial infarction. Heart Lung.

[38] West R.R., Jones D.A., Henderson A.H. (2012). Rehabilitation after myocardial infarction trial (RAMIT): Multi-centre randomised controlled trial of comprehensive cardiac rehabilitation in patients following acute myocardial infarction. Heart.

[39] Wienbergen H., Fach A., Meyer S., Meyer J., Stehmeier J., Backhaus T., Michel S., Krämer K., Osteresch R., Schmucker J. (2019). Effects of an intensive long-term prevention programme after myocardial infarction—A randomized trial. Eur. J. Prev. Cardiol..

[40] Yousefy A., Keshtiaray N., Yamani N., Rabiei K., Baghbanian P. (2009). Quality of life in post myocardial infarction patients with or without cardiac rehabilitation. Res. J. Biol. Sci..

[41] Johnson N.A., Lim L.L.Y., Bowe S.J. (2009). Multicenter randomized controlled trial of a home walking intervention after outpatient cardiac rehabilitation on health-related quality of life in women. Eur. J. Cardiovasc. Prev. Rehabil..

[42] Hurdus B., Munyombwe T., Dondo T.B., Aktaa S., Oliver G., Hall M., Doherty P., Hall A.S., Gale C.P., Hurdus B. (2020). Association of cardiac rehabilitation and health-related quality of life following acute myocardial infarction. Heart.

[43] Aldana S.G., Whitmer W.R., Greenlaw R., Avins A.L., Salberg A., Barnhurst M., Fellingham G., Lipsenthal L. (2003). Cardiovascular risk reductions associated with aggressive lifestyle modification and cardiac rehabilitation. Heart Lung.

[44] Chan D.S.K., Chau J.P.C., Chang A.M. (2005). Acute coronary syndromes: Cardiac rehabilitation programmes and quality of life. J. Adv. Nurs..

[45] Taylor R.S., Brown A., Ebrahim S., Jolliffe J., Noorani H., Rees K., Skidmore B., Stone J.A., Thompson D.R., Oldridge N. (2004). Exercise-based rehabilitation for patients with coronary heart disease: Systematic review and meta-analysis of randomized controlled trials. Am. J. Med..

[46] Anderson L., Sharp G.A., Norton R.J., Dalal H., Dean S.G., Jolly K., Cowie A., Zawada A., Taylor R.S. (2017). Home-based versus centre-based cardiac rehabilitation. Cochrane Database Syst. Rev..

[47] Shibata A., Oka K., Nakamura Y., Muraoka I. (2007). Recommended level of physical activity and health-related quality of life among Japanese adults. Health Qual. Life Outcomes.

[48] Wendel-Vos G.C.W., Schuit A.J., Tijhuis M.A.R., Kromhout D. (2004). Leisure time physical activity and health-related quality of life: Cross-sectional and longitudinal associations. Qual. Life Res..

[49] Morimoto T., Oguma Y., Yamazaki S., Sokejima S., Nakayama T., Fukuhara S. (2006). Gender differences in effects of physical activity on quality of life and resource utilization. Qual. Life Res..

[50] Chen Y.W., Wang C.Y., Lai Y.H., Liao Y.C., Wen Y.K., Chang S.T., Huang J.L., Wu T.J. (2018). Home-based cardiac rehabilitation improves quality of life, aerobic capacity, and readmission rates in patients with chronic heart failure. Medicine.

[51] Haykowsky M., Scott J., Esch B., Schopflocher D., Myers J., Paterson I., Warburton D., Jones L., Clark A.M. (2011). A meta-analysis of the effects of exercise training on left ventricular remodeling following myocardial infarction: Start early and go longer for greatest exercise benefits on remodeling. Trials.

[52] Parker K., Stone J.A., Arena R., Lundberg D., Aggarwal S., Goodhart D., Traboulsi M. (2011). An early cardiac access clinic significantly improves cardiac rehabilitation participation and completion rates in low-risk ST-elevation myocardial infarction patients. Can. J. Cardiol..

[53] Haeny T., Nelson R., Ducharme J., Zuhl M. (2019). The influence of exercise workload progression across 36 Sessions of cardiac rehabilitation on functional capacity. J. Cardiovasc. Dev. Dis..

[54] Linden W. (2000). Psychological treatments in cardiac rehabilitation: Review of rationales and outcomes. J. Psychosom. Res..

[55] Dickens C.M., McGowan L., Percival C., Tomenson B., Cotter L., Heagerty A., Creed F.H. (2006). Contribution of depression and anxiety to impaired health-related quality of life following first myocardial infarction. Br. J. Psychiatry.

[56] Palermi S., Bragazzi N., Cular D., Ardigò L., Padulo J. (2022). How chest press-based exercises can alleviate the burden of cardiovascular diseases. Hum. Mov..

[57] Palermi S., Sacco A.M., Belviso I., Romano V., Montesano P., Corrado B., Sirico F. (2020). Guidelines for Physical Activity—A Cross-Sectional Study to Assess Their Application in the General Population. Have We Achieved Our Goal?. Int. J. Environ. Res. Public Health.

[58] Rumsfeld J., Alexander K., Goff D., Graham M., Ho P., Masoudi F., Moser D., Roger V., Slaughter M., Smolderen K. (2013). Cardiovascular health: The importance of measuring patient-reported health status: A scientific statement from the American Heart Association. Circulation.

[59] Lavie C.J., Arena R., Franklin B.A. (2016). Cardiac rehabilitation and healthy life-style interventions rectifying programme deficiencies to improve patient outcomes. J. Am. Coll. Cardiol..

[60] Jolly K., Taylor R.S., Lip G.Y.H., Stevens A. (2006). Home-based cardiac rehabilitation compared with centre-based rehabilitation and usual care: A systematic review and meta-analysis. Int. J. Cardiol..

[61] Gutenbrunner C., Stievano A., Nugraha B., Stewart D., Catton H. (2021). Nursing—A core element of rehabilitation. Int. Nurs. Rev..

[62] Dithmer M., Rasmussen J.O., Grönvall E., Spindler H., Hansen J., Nielsen G., Sørensen S.B., Dinesen B. (2016). “The Heart Game”: Using gamification as part of a telerehabilitation programme for heart patients. Games Health J..

[63] Vidal-Almela S., Czajkowski B., Prince S.A., Chirico D., Way K.L., Pipe A.L., Reed J.L. (2020). Lessons learned from community- and home-based physical activity programmes: A narrative review of factors influencing women’s participation in cardiac rehabilitation. Eur. J. Prev. Cardiol..

