# Supplementary figures and images for: Effects of Supervised Cardiac Rehabilitation Programmes on Quality of Life among Myocardial Infarction Patients: A Systematic Review and Meta-Analysis
Source: J Cardiovasc Dev Dis. 2021 Nov 27;8(12):166. doi: 10.3390/jcdd8120166 (PMC8703932; doi:10.3390/jcdd8120166)

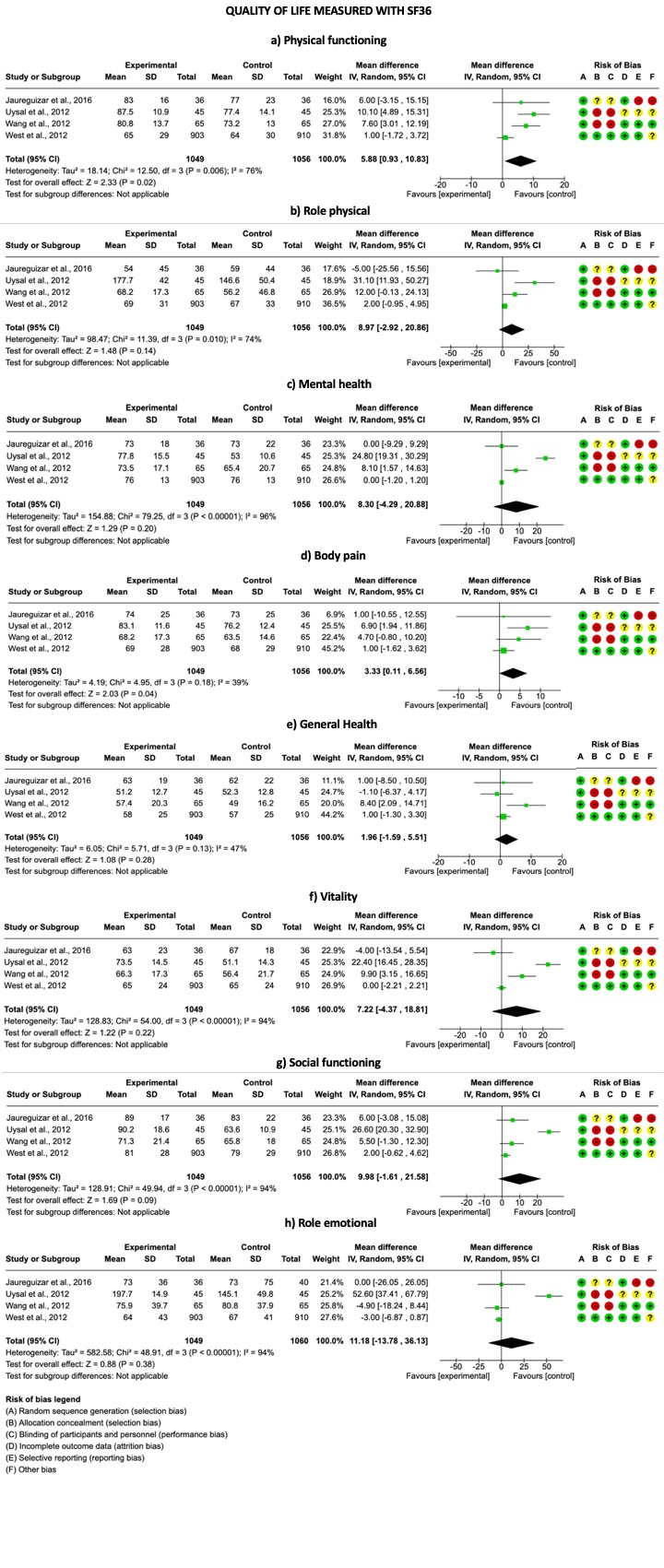

Supplement: Supplementary file 1 [file jcdd-08-00166-s001.zip › Figure S1. SF-36.jpg]

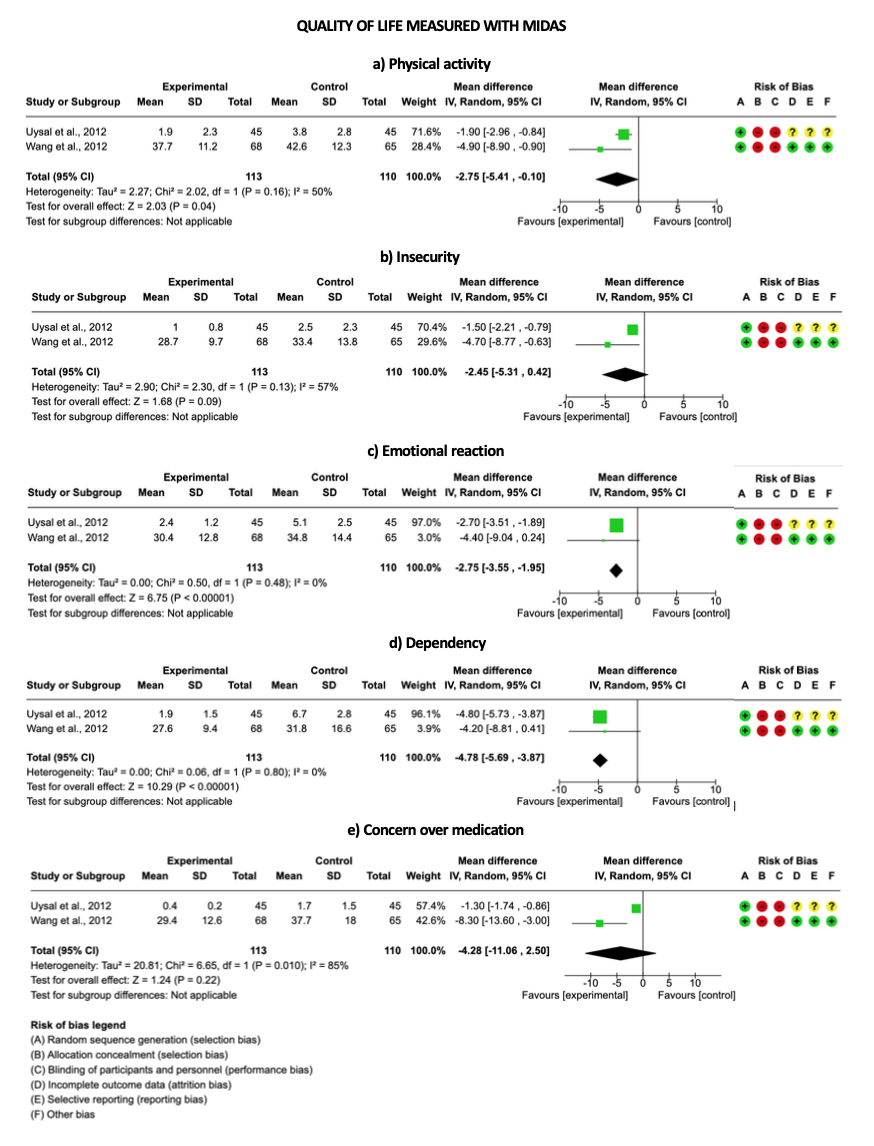

Supplement: Supplementary file 1 [file jcdd-08-00166-s001.zip › Figure S2. MIDAS.jpg]

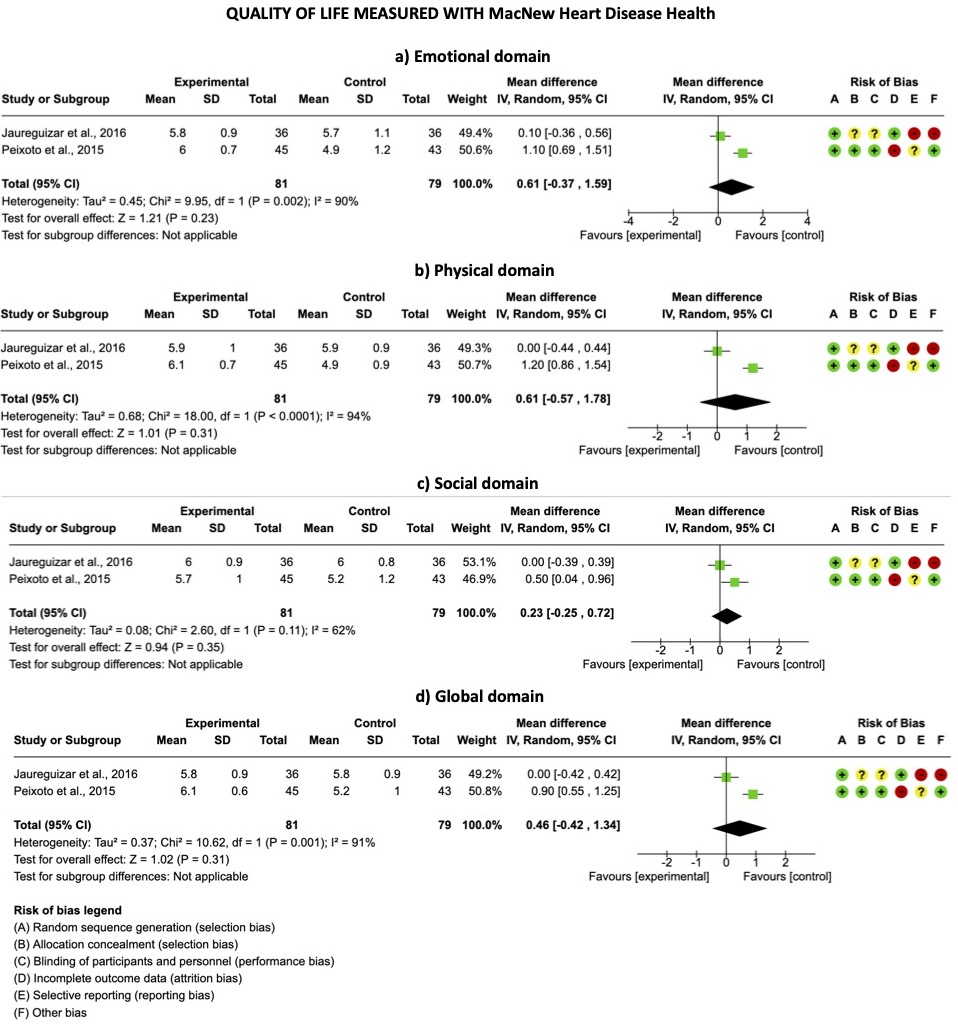

Supplement: Supplementary file 1 [file jcdd-08-00166-s001.zip › Figure S3. MacNew.jpg]

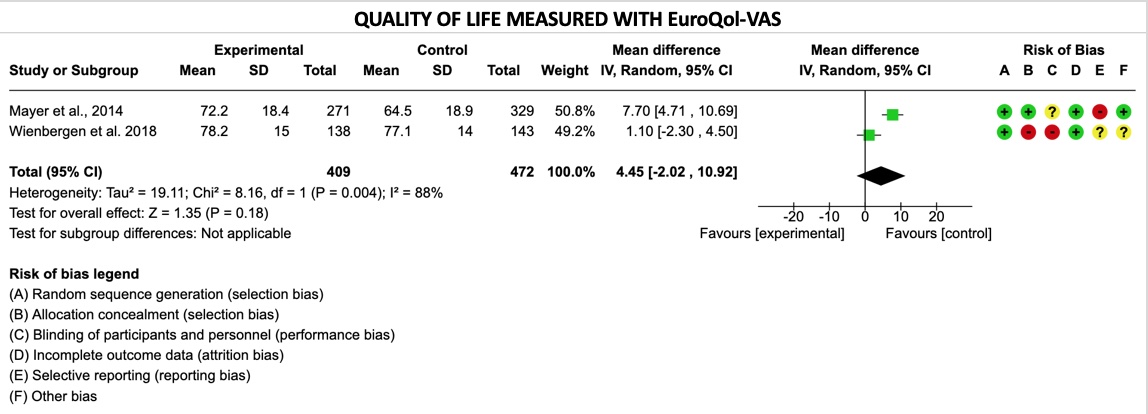

Supplement: Supplementary file 1 [file jcdd-08-00166-s001.zip › Figure S4. EuroQol-VAS.jpg]
